# Congruency sequence effects are driven by previous-trial congruency, not previous-trial response conflict

**DOI:** 10.3389/fpsyg.2013.00587

**Published:** 2013-09-04

**Authors:** Daniel H. Weissman, Joshua Carp

**Affiliations:** Department of Psychology, University of Michigan, Ann Arbor, MI, USA

**Keywords:** congruency sequence effects, conflict monitoring, reaction time, face-word Stroop, sequential modulations

## Abstract

Congruency effects in distracter interference tasks are often smaller after incongruent trials than after congruent trials. However, the sources of such congruency sequence effects (CSEs) are controversial. The conflict monitoring model of cognitive control links CSEs to the detection and resolution of response conflict. In contrast, competing theories attribute CSEs to attentional or affective processes that vary with previous-trial congruency (incongruent vs. congruent). The present study sought to distinguish between conflict monitoring and congruency-based accounts of CSEs. To this end, we determined whether CSEs are driven by previous-trial reaction time (RT)—a putative measure of response conflict—or by previous-trial congruency. In two experiments using a face-word Stroop task (*n* = 49), we found that current-trial congruency effects did not vary with previous-trial RT independent of previous-trial congruency. In contrast, current-trial congruency effects *were* influenced by previous-trial congruency independent of previous-trial RT. These findings appear more consistent with theories that attribute CSEs to non-conflict processes whose recruitment varies with previous-trial congruency than with theories that link CSEs to previous-trial response conflict.

## Introduction

First observed in a flanker task two decades ago (Gratton et al., 1992), congruency sequence effects (CSEs) refer to smaller congruency effects after incongruent trials than after congruent trials in distracter interference tasks. Since that time, CSEs, also called Gratton effects or conflict adaptation (Botvinick et al., 2001), have been observed in the flanker task (Ullsperger et al., 2005), the Simon task (Wühr, 2005), and Stroop and Stroop-like tasks (Kerns et al., 2004; Notebaert and Verguts, 2007; Egner et al., 2010). While early observations of CSEs may have been driven by confounds between congruency sequence and stimulus or response repetition (Mayr et al., 2003; Hommel et al., 2004), recent studies have documented CSEs while rigorously controlling for such confounds (Egner et al., 2010; Akçay and Hazeltine, 2011; Compton et al., 2012); (for a review, see Egner, 2008). Nonetheless, the psychological mechanisms underlying CSEs remain controversial.

The conflict monitoring model posits that CSEs stem from variations of previous-trial response conflict (i.e., the simultaneous activation of competing responses) (Botvinick et al., 2001; Yeung et al., 2004). In particular, the model posits that heightened response conflict in the previous trial triggers increased attention to task-relevant stimuli and responses in the current trial. The model also posits that reaction time (RT) is a more direct index of response conflict than congruency because “conflict more closely tracks RT than congruence condition when the two are dissociated” (Yeung et al., 2011, p. 3). Further, although the model acknowledges the existence of processes other than response conflict (e.g., attention, response preparation, etc.), it appears to hold that such processes affect RT only through their influence on response conflict. As explained by Yeung et al. (2011).

“… slow congruent trials are not slow *despite* having low conflict, and fast incongruent trials are not fast *despite* having high conflict. To the contrary, slow congruent trials are slow precisely *because* conflict is high—a consequence of failing to focus attention, misperceiving the distracter, or preparing the wrong response, etc.—while fast incongruent trials are fast *because* conflict is low (Yeung et al., 2011, p. 3–4).”

Given that variations of RT are mediated solely through variations of response conflict, the model posits that incongruent and congruent trials with equivalent RTs are associated with equivalent response conflict and that, within any particular trial type, slow-RT trials are associated with greater response conflict than fast-RT trials (Yeung et al., 2011). In short, the model posits that CSEs are driven by response conflict, which is directly indexed by trial-specific RT.

In contrast, competing theories posit that CSEs are driven by attentional or affective processes that vary with previous-trial congruency. For example, participants may develop expectations about current-trial congruency based on previous-trial congruency and, consequently, allocate more or less attention to the current-trial distracter (Gratton et al., 1992). Participants may also experience congruency switch costs while alternating between processes that underlie the processing of incongruent and congruent stimuli; these switch costs then manifest as CSEs (Schmidt and De Houwer, 2011). Finally, participants may experience greater negative affect when presented with incongruent stimuli relative congruent stimuli, triggering increased attention to task-relevant stimuli and responses in the next trial (van Steenbergen et al., 2010, 2012; Dreisbach and Fischer, 2011, 2012). Despite extensive study, the debate between conflict monitoring and congruency-based accounts of CSEs remains unresolved.

The present study sought to resolve this controversy by capitalizing on the fact that conflict monitoring and congruency-based accounts of CSEs make distinct predictions about the influences of previous-trial RT and previous-trial congruency on current-trial congruency effects. As described earlier, the conflict monitoring model posits that RT is a direct index of response conflict (Yeung et al., 2011). Coupled with the model's assertion that response conflict drives CSEs (Botvinick et al., 2001), this assumption leads the model to predict that current-trial congruency effects should be (a) equivalent after RT-matched incongruent and congruent trials and (b) smaller after slow-RT than after fast-RT trials within any particular trial type. Put simply, the model predicts an influence of previous-trial RT on current-trial congruency effects that is independent of previous-trial congruency. In contrast, congruency-based accounts posit that CSEs are driven by attentional or affective processes related to previous-trial congruency. Thus, such accounts predict a relationship between previous-trial congruency and current-trial congruency effects that is independent of previous-trial RT.

We investigated these competing predictions in two experiments. To anticipate, we found that current-trial congruency effects were not influenced by previous-trial RT independent of previous-trial congruency but *were* influenced by previous-trial congruency independent of previous-trial RT. These results appear more consistent with the predictions of congruency-based accounts of CSEs than with the predictions of the conflict monitoring model.

## Experiment 1

### Methods

#### Participants

Twenty-three healthy adults (mean age: 20.4 years; age range: 18–29 years; 16 females) from the University of Michigan community participated in the experiment. All gave written informed consent prior to participating in accordance with the University of Michigan's Institutional Review Board. Each individual was paid $10 per h for his or her participation.

#### Stimuli

Face-word stimuli in the form of black and white photographs from Egner et al. (2010) were provided by Tobias Egner. There were 12 males and 12 females faces. We used Presentation software (Neurobehavioral Systems, www.neurobs.com) on Dell Vostro PCs running Windows XP to present the task stimuli and to record participants' responses.

#### Task and procedure

**Figure 1:**
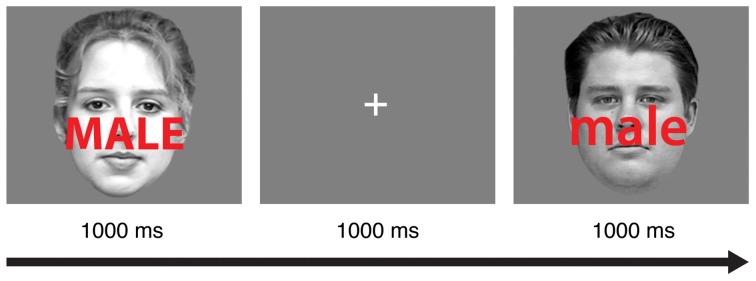
**A schematic of the experimental task.** In each trial, subjects were instructed to discriminate the gender of the face image (male or female) while ignoring the gender of the superimposed word (male or female). In this sample trial sequence, an incongruent stimulus in one trial (left) is followed by a congruent stimulus in the subsequent trial (right).

Participants completed 16 practice trials with auditory feedback after each incorrect response. Next, they completed eight 64-trial blocks of a face-word Stroop task adapted from a recent study (Egner et al., 2010). In each trial (Figure 1), participants viewed a male or female face with a superimposed distracter word (“male” or “female”) for 1000 ms. Participants were instructed to identify the gender of the face as quickly as possible without making mistakes while ignoring the gender of the word. In congruent trials, the genders of the face and the word matched (e.g., a female face presented with the word “female”). In incongruent trials, the genders of the face and word were different (e.g., a female face presented with the word “male”). Participants indicated the gender of the face (male or female) using the “j” and “k” keys on a standard keyboard, and the gender-response mapping was counterbalanced across participants. A 1000 ms interval was provided for participants to respond.

A 2000 ms stimulus onset asynchrony separated adjacent trials. As in Egner et al. (2010), we did not allow exact repetitions of the face or the word across consecutive trials. Specifically, no face repeated in consecutive trials, and the word's case (upper or lower) alternated every trial. In all other respects, the stimulus construction procedure for creating face-word pairings was randomized.

#### Data analysis

Prior to analyzing the data from each participant, we removed trials in which no response was made and trials with RTs greater than three standard deviations from the condition-specific mean. For analyses of the RT data, we also removed error trials and trials immediately following errors. For analyses of the accuracy data, error trials and trials immediately following errors were retained.

To control for the effect of previous-trial RT on CSEs, we created a subset of congruent and incongruent trials with equivalent RTs for each participant (Carp et al., 2010). For each congruent trial, we identified an incongruent trial whose RT fell within 5 ms of the congruent-trial RT. If multiple incongruent trials met this criterion, then the incongruent trial with the RT closest to the congruent-trial RT was chosen. Trials that could not be matched across conditions were discarded from this analysis. After the RT-matched data set was created, we determined whether current-trial congruency effects differed after RT-matched congruent and incongruent trials. Current congruent and current incongruent trials were not limited to those in the RT-matched data set (i.e., they were drawn from the full data set).

We also investigated the relationship between previous-trial RT and current-trial congruency effects. To do so, we identified the 33% slowest trials and the 33% fastest trials in each trial type (incongruent and congruent). We then asked whether current-trial congruency effects differed after the 33% slowest vs. the 33% fastest (a) incongruent trials and (b) congruent trials. As in the RT-matching analysis above, current congruent and current incongruent trials in the RT tertile analysis were not limited to the 33% fastest and 33% slowest trials in each trial type (i.e., they were drawn from the full data set).

### Results

#### Congruency sequence effects in the full data set

As in previous studies of the face-word Stroop task (Egner et al., 2008, 2010), mean RT was longer in incongruent than in congruent trials [528 vs. 514 ms; *t*_(22)_ = 3.66, *p* = 0.001]. Analogously, mean accuracy was lower in incongruent than in congruent trials [94.9 vs. 97.0%; *t*_(22)_ = 3.64, *p* = 0.001]. Thus, effects of congruency on RT did not stem from speed-accuracy tradeoffs.

**Figure 2:**
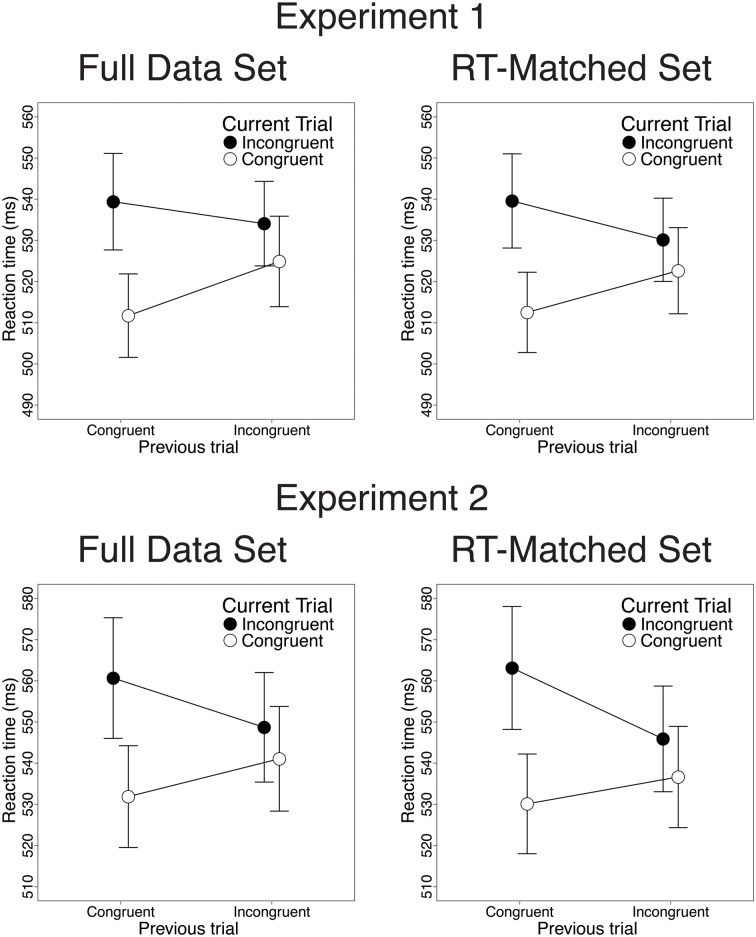
**Congruency sequence effects (CSEs) in the RT data.** We observed robust CSEs in the full data set [Experiment 1: *t*_(22)_ = 3.82, *p* < 0.001; Experiment 2: *t*_(25)_ = 5.32, *p* < 0.001] and in the RT-matched data set [Experiment 1: *t*_(22)_ = 4.34, *p* < 0.001; Experiment 2: *t*_(25)_ = 5.61, *p* < 0.001]. CSEs did not differ across these data sets in Experiment 1 [*t*_(22)_ = 0.51, *n.s*.], and were non-significantly larger in the RT-matched data set than in the full data set in Experiment 2 [*t*_(25)_ = 2.01, *p* = 0.055]. Error bars indicate the standard error of the mean.

**Figure 3:**
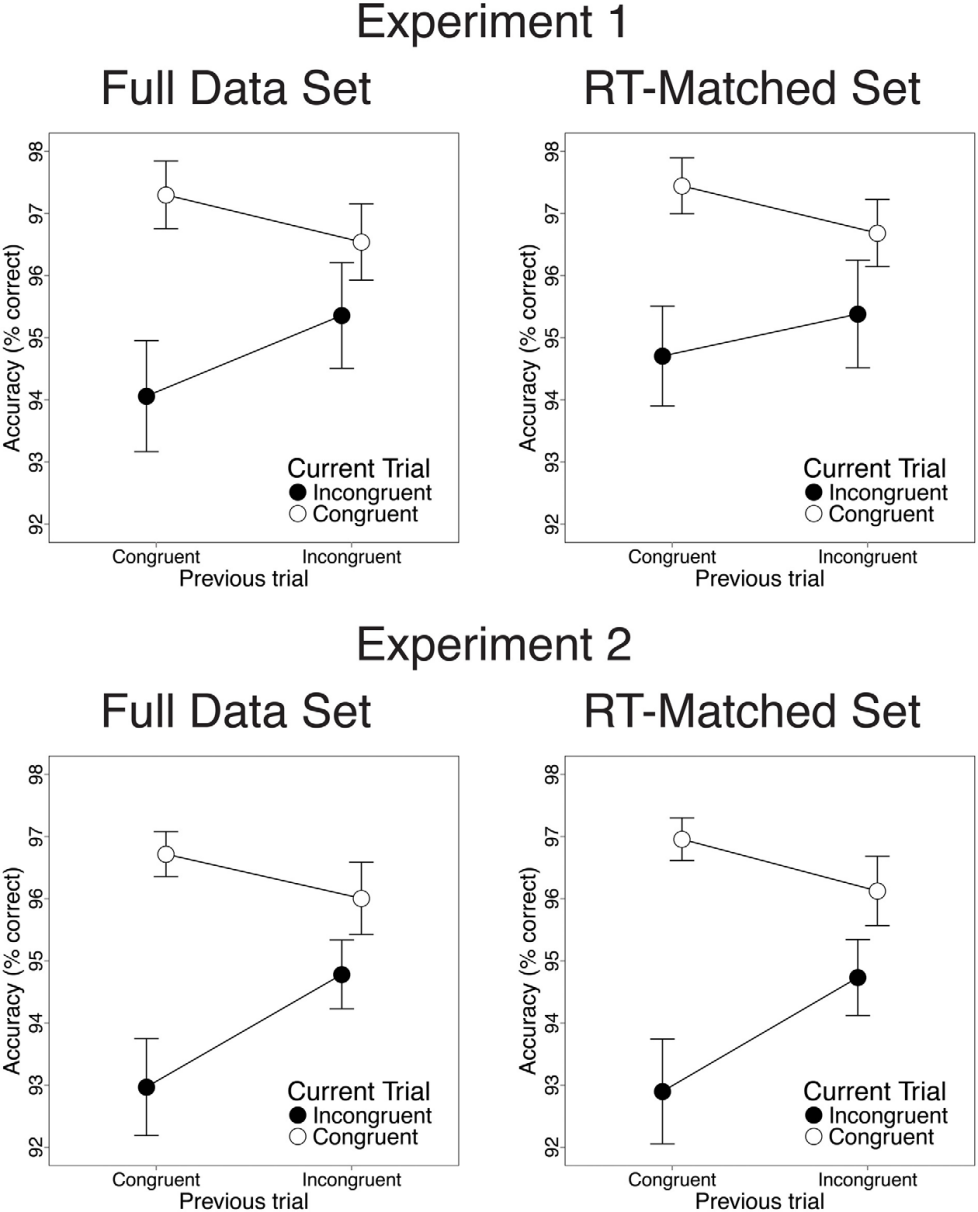
**Congruency sequence effects (CSEs) in the accuracy data.** We observed significant CSEs in the full data set [Experiment 1: *t*_(22)_ = 2.47, *p* = 0.02; Experiment 2: *t*_(25)_ = 2.91, *p* = 0.007] and in the RT-matched data set [Experiment 1: *t*_(22)_ = 1.94, *p* = 0.065; Experiment 2: *t*_(25)_ = 3.27, *p* = 0.003]. CSEs did not differ across data sets [Experiment 1: *t*_(22)_ = 1.23, *n.s*.; Experiment 2: *t*_(25)_ = 0.38, *n.s*.]. Error bars indicate the standard error of the mean.

Also consistent with previous studies, congruency effects on both RT and response accuracy were reduced after incongruent trials, relative to congruent trials. In the RT data, we observed greater congruency effects after congruent than after incongruent trials [28 vs. 9.2 ms; *t*_(22)_ = 3.82, *p* < 0.001; Figure 2, upper left]. This CSE was driven by faster congruent-trial RT after congruent than after incongruent trials [13.2 ms; *t*_(22)_ = 5.33, *p* < 0.001] and by a trend toward faster incongruent-trial RT after incongruent than after congruent trials [5.3 ms; *t*_(22)_ = 1.39, *n.s*.]. In the accuracy data, congruency effects were also larger after congruent than after incongruent trials [3.2% vs. 1.2%; *t*_(22)_ = 2.47, *p* = 0.02; Figure 3, upper left]. This CSE was driven by non-significantly greater congruent-trial accuracy after congruent than after incongruent trials [0.76%; *t*_(22)_ = 1.58, *n.s.*] and by significantly greater incongruent-trial accuracy after incongruent than after congruent trials [1.3%; *t*_(22)_ = 2.04, *p* = 0.054].

#### Congruency effects after RT-matched congruent and incongruent trials

Next, we investigated whether CSEs were smaller after RT-matched congruent and incongruent trials than in the full data set. An RT-matching procedure (see Methods) allowed us to select congruent and incongruent trials that were naturally matched for RT. On average, the RT-matching procedure removed 22.8% of trials, leaving an average of 169.1 pairs of RT-matched congruent and incongruent trials in each participant. As intended, mean RT for incongruent and congruent trials did not differ in the RT-matched data set [514 vs. 514 ms; *t*_(22)_ = 1.20, *n.s*.]. However, mean accuracy remained lower in incongruent than in congruent trials [95.0 vs. 97.4%; *t*_(22)_ = 3.44, *p* = 0.002].

Contrary to the predictions of the conflict monitoring model, but consistent with congruency-based accounts of CSEs, we observed statistically indistinguishable CSEs before and after RT-matching. For the RT data, CSEs did not differ between the RT-matched and full data sets [20 vs. 18 ms; *t*_(22)_ = 0.51, *n.s*.]. As in the full data set, the RT-matched data set yielded greater congruency effects after congruent trials than after incongruent trials [27 vs. 7.5 ms; *t*_(22)_ = 4.34, *p* < 0.001; Figure 2, upper right]. This CSE was driven by faster congruent-trial RT after congruent than after incongruent trials [10.1 ms; *t*_(22)_ = 3.96, *p* < 0.001] and by faster incongruent-trial RT after incongruent trials than after congruent trials [9.5 ms; *t*_(22)_ = 2.24, *p* = 0.035]. For the accuracy data, we also found equivalent CSEs in the RT-matched full data sets [1.4 vs. 2.1%; *t*_(22)_ = 1.23, *n.s*.]. In the RT-matched data set, we observed a trend toward larger congruency effects after congruent than after incongruent trials [2.7 vs. 1.3%; *t*_(22)_ = 1.94, *p* = 0.065; Figure 3, upper right]. This trend toward a CSE was driven by numerically greater congruent-trial accuracy after congruent than after incongruent trials [0.76%; *t*_(22)_ = 1.54, *n.s*.], and by non-significantly greater incongruent-trial accuracy after incongruent than after congruent trials [0.68%; *t*_(22)_ = 1.01, *n.s*.].

#### Congruency effects after slow-RT vs. fast-RT trials

**Figure 4:**
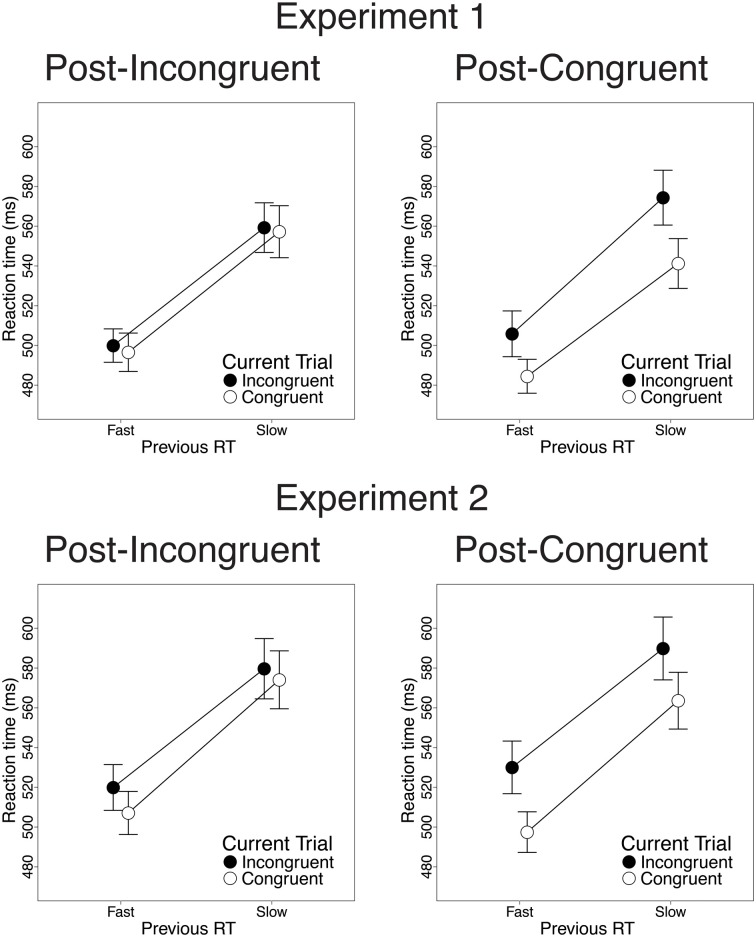
**The effects of previous-trial RT tertile on current-trial congruency effects in the RT data.** Congruency effects did not differ after the 33% slowest vs. the 33% fastest incongruent trials [Experiment 1: *t*_(22)_ = 0.20, *n.s*.; Experiment 2: *t*_(25)_ = 1.51, *n.s*.] or congruent trials [Experiment 1: *t*_(22)_ = −1.8, *n.s*.; Experiment 2: *t*_(25)_ = 1.10, *n.s*.]. Error bars indicate the standard error of the mean.

**Figure 5:**
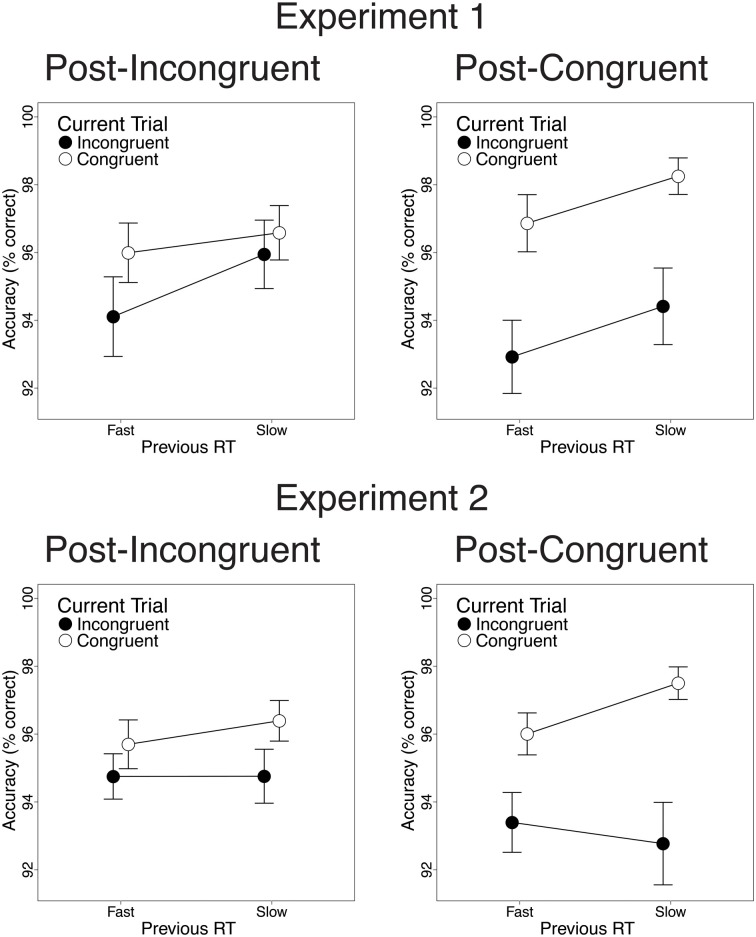
**The effects of previous-trial RT tertile on current-trial congruency effects in the accuracy data.** Congruency effects did not differ after the 33% slowest vs. the 33% fastest incongruent trials [Experiment 1: *t*_(22)_ = 0.91, *n.s*.; Experiment 2: *t*_(25)_ = 0.70, *n.s*.] or congruent trials [Experiment 1: *t*_(22)_ = 0.11, *n.s*.; Experiment 2: *t*_(25)_ = 1.8, *n.s*.]. Error bars indicate the standard error of the mean.

Finally, we investigated the relationship between previous-trial RT and current-trial congruency effects. Specifically, we divided correct incongruent and correct congruent trials into RT tertiles (see Methods) and determined the magnitude of current-trial congruency effects following (a) the 33% slowest vs. 33% fastest incongruent trials and (b) the 33% slowest vs. 33% fastest congruent trials. As intended, mean RT was longer in slow-RT than fast-RT incongruent trials [606 vs. 430 ms; *t*_(22)_ = 24.2, *p* < 0.001] and congruent trials [630 vs. 437 ms; *t*_(22)_ = 26.6, *p* < 0.001]. Critically, contrary to the predictions of the conflict monitoring model, but consistent with congruency-based accounts of CSEs, previous-trial RT tertile had no significant impact on current-trial congruency effects. In the RT data, we observed equivalent congruency effects after slow-RT vs. fast-RT incongruent trials [2.1 vs. 3.4 ms; *t*_(22)_ = 0.20, *n.s*.; Figure 4, upper left] and congruent trials [33 vs. 21 ms; *t*_(22)_ = −1.8, *n.s*.; Figure 4, upper right]. In the accuracy data, we also found comparable congruency effects following slow-RT vs. fast-RT incongruent trials [1.9 vs. 0.64%; *t*_(22)_ = 0.91, *n.s*.; Figure 5, upper left] and congruent trials [3.9 vs. 3.8%; *t*_(22)_ = 0.11, *n.s*.; Figure 5, upper right].

### Discussion

In Experiment 1, we evaluated the competing predictions of conflict monitoring and congruency-based accounts of CSEs. The conflict monitoring model predicts an influence of previous-trial RT on current-trial congruency effects that is independent of previous-trial congruency. In contrast, congruency-based accounts predict an influence of previous-trial congruency on current-trial congruency effects that is independent of previous-trial RT. Our findings appeared to support congruency-based accounts, in which CSEs are driven by previous-trial congruency.

However, the design of Experiment 1 suffered from two limitations. First, because the order of trial conditions was randomized, confounds between congruency sequence and response sequence could have contributed to CSEs in the full data set (Egner, 2007). Second, given our random procedure for pairing faces with words, some face-word pairings may have been presented more often than others, and this effect may have differed between congruent and incongruent trials. Critically, any such difference could have led to contingency biases that can inflate estimates of CSEs (Schmidt and De Houwer, 2011). We conducted Experiment 2 to rule out these potential confounds.

## Experiment 2

### Methods

#### Participants

As in Experiment 1, participants were recruited using flyers posted on the University of Michigan campus. Experiment 2 included 26 participants (17 females), with an average age of 19.4 years and an age range of 18–22 years. As in Experiment 1, informed written consent was obtained from each participant, and participants were paid $10 per h.

#### Stimuli

The face images and gender words were identical to those used in Experiment 1.

#### Task and procedure

The task and procedure were identical in most respects to those used in Experiment 1 with three exceptions. First, in each block, the four possible combinations of face gender (male or female) and word gender (male or female) were first-order counterbalanced across trials. Thus, each possible face-word combination was preceded equally often by each of the four types of face-word combinations. This constraint yielded equal frequencies of occurrence for the four possible first-order congruency sequences (i.e., congruent-congruent, incongruent-congruent, congruent-incongruent, and incongruent-incongruent). It also ensured that response repetitions and response alternations occurred equally often in each of these congruency sequences. Second, each of the 24 actors (12 males, 12 females) was paired equally often with each gender word (“female” and “male”) and each case (upper and lower). This constraint ensured that each face-word pairing occurred equally often, thereby ruling out contingency biases that can lead to spurious CSEs (Schmidt and De Houwer, 2011). Third, participants completed 24 practice trials and eight blocks of 96 trials during the experiment.

#### Data analysis

The data analysis was identical to that in Experiment 1.

### Results

#### Congruency sequence effects in the full data set

As in Experiment 1, mean RT was longer in incongruent than in congruent trials [546 vs. 528 ms; *t*_(25)_ = 5.20, *p* < 0.001]. Mean accuracy was lower in incongruent than in congruent [94.2 vs. 96.6%] trials [*t*_(25)_ = 6.49, *p* < 0.001].

Also paralleling the results of Experiment 1, we observed significant CSEs for both RT and accuracy. In the RT data, congruency effects were larger after congruent than after incongruent trials [28.8 vs. 7.6 ms; *t*_(25)_ = 5.32, *p* < 0.001; Figure 2, lower left]. This CSE was driven by faster congruent-trial RT after congruent than after incongruent trials [9.2 ms; *t*_(25)_ = 3.44, *p* = 0.002] and by faster incongruent-trial RT after incongruent than after congruent trials [12 ms; *t*_(25)_ = 3.27, *p* = 0.003]. In the accuracy data, we also observed larger congruency effects after congruent than after incongruent trials [3.7 vs. 1.2%; *t*_(25)_ = 2.91, *p* = 0.007; Figure 3, lower left]. This CSE was driven by a trend toward higher congruent-trial accuracy after congruent than after incongruent trials [0.71%; *t*_(25)_ = 1.54; *n.s*.] and by significantly higher incongruent-trial accuracy after incongruent than after congruent trials [1.8%; *t*_(25)_ = 3.17; *p* = 0.004].

#### Congruency effects after RT-matched congruent and incongruent trials

We next investigated whether CSEs after RT-matched congruent and incongruent trials were smaller than CSEs in the full data set. The RT-matching procedure removed 19.5% of trials on average, leaving an average of 263.0 pairs of RT-matched congruent and incongruent trials for each participant. As intended, mean RT for incongruent and congruent trials in the RT-matched data set was equivalent [532 vs. 532 ms; *t*_(25)_ = 0.46, *n.s*.]. However, as in Experiment 1, mean accuracy remained lower in incongruent than in congruent trials [94.6 vs. 97.1%; *t*_(25)_ = 5.55, *p* < 0.001].

Contrary to the predictions of the conflict monitoring model, but consistent with congruency-based accounts of CSEs, we observed statistically indistinguishable CSEs before and after RT-matching. For the RT data, CSEs did not differ across the RT-matched and full data sets [24vs. 21 ms; *t*_(25)_ = 2.01, *p* = 0.055]. In the RT-matched data set, we observed larger congruency effects after congruent trials than after incongruent trials [33 vs. 9.3 ms; *t*_(25)_ = 5.61, *p* < 0.001; Figure 2, lower right]. This CSE reflected faster congruent-trial RT after congruent than after incongruent trials [6.5 ms; *t*_(25)_ = 2.77, *p* = 0.01], as well as faster incongruent-trial RT after incongruent than after congruent trials [17 ms; *t*_(25)_ = 4.32, *p* < 0.001]. For the accuracy data, CSEs were also comparable in the RT-matched and full data sets [2.7 vs. 2.5%; *t*_(25)_ = 0.38, *n.s*.]. In the RT-matched data set, congruency effects were larger after congruent than after incongruent trials [4.1 vs. 1.4%; *t*_(25)_ = 3.27, *p* = 0.003; Figure 3, lower right]. This CSE reflected both a trend toward greater congruent-trial accuracy after congruent than after incongruent trials [0.83%; *t*_(25)_ = 1.77, *p* = 0.089] and significantly greater incongruent-trial accuracy after incongruent than after congruent trials [1.8%; *t*_(25)_ = 2.95, *p* = 0.0067].

#### Congruency effects after slow-RT vs. fast-RT trials

Finally, we investigated the relationship between previous-trial RT and current-trial congruency effects by dividing correct incongruent and correct congruent trials into RT tertiles (see Methods). As intended, mean RT was longer in the 33% slowest trials than in the 33% fastest trials for both incongruent trials [658 vs. 447 ms; *t*_(25)_ = 20.1, *p* < 0.001] and congruent trials [630 vs. 439 ms; *t*_(25)_ = 19.4, *p* < 0.001]. Contrary to the conflict monitoring model, but consistent with congruency-based accounts of CSEs, we observed no relationship between previous-trial RT and current-trial congruency effects. In the RT data, current-trial congruency effects did not differ after slow-RT vs. fast-RT incongruent trials [5.6 vs. 12.2 ms; *t*_(25)_ = 1.51, *n.s*.; Figure 4, lower left] or congruent trials [26 vs. 33 ms; *t*_(25)_ = 1.10, *n.s*.; Figure 4, lower right]. In the accuracy data, congruency effects also did not differ after slow-RT vs. fast-RT incongruent trials [0.95 vs. 1.6%; *t*_(25)_ = 0.70, *n.s*.; Figure 5, lower left] or congruent trials [2.6 vs. 4.7%; *t*_(25)_ = 1.8, *n.s*.; Figure 5, lower right].

### Discussion

Experiment 2 replicated the key results of Experiment 1 and therefore provided stronger support for congruency-based accounts of CSEs. First, we observed robust CSEs in the full data set, and these effects remained significant in the RT-matched data set. Second, current-trial congruency effects did not differ after slow-RT vs. fast-RT incongruent trials or after slow-RT vs. fast-RT congruent trials. Finally, Experiment 2 ruled out the possibility that CSEs in the full data set were due to confounds between congruency sequence and response sequence (Hommel et al., 2004; Nieuwenhuis et al., 2006) or contingency biases (Schmidt and De Houwer, 2011).

## General discussion

The present study sought to distinguish between the conflict monitoring model and congruency-based accounts of CSEs. As predicted by congruency-based accounts, current-trial congruency effects were influenced by previous-trial congruency independent of previous-trial RT. However, inconsistent with the conflict monitoring model, current-trial congruency effects were not influenced by previous-trial RT—a putative index of response conflict—independent of previous-trial congruency (Yeung et al., 2011). These findings provide novel support for congruency-based accounts of CSEs but pose a challenge to the conflict monitoring model. They also bolster prior studies suggesting that CSEs might not be driven by response conflict (Verbruggen et al., 2006; Lamers and Roelofs, 2011; Compton et al., 2012).

Our findings suggest that the conflict monitoring model suffers from at least one of two potential limitations. First, if the model's assumption that RT serves as a direct index of response conflict is correct (Yeung et al., 2011), then our finding that current-trial congruency effects are not influenced by previous-trial RT is inconsistent with the model's claim that response conflict drives CSEs. The second potential limitation is that trial-specific RT may not directly index response conflict. If this is the case, then the present results would not challenge the view that CSEs are driven by previous-trial response conflict. However, they would indicate a need to revise the model so that RT and response conflict can be decoupled. Consistent with the need for such a decoupling, prior findings from EEG indicate that congruency effects are absent after correctly-performed trials in which a competing response was activated (i.e., when response conflict was likely) but present after correctly-performed trials in which a competing response was not activated (i.e., when response conflict was unlikely), independent of RT variability within each of these “previous” trial types (Burle et al., 2002).

One might argue that it is not a problem for the model if RT does not directly (and solely) index response conflict. Indeed, RT could also reflect a variety of non-conflict processes (e.g., attention, motivation, etc.). As described in the Introduction, however, the model does not appear to permit variations of RT independent of response conflict. Further, Yeung and colleagues (Yeung et al., 2011) have invoked this feature of the model to explain recent observations that RT-matched incongruent and congruent trials elicit equivalent activation in the posterior medial frontal cortex, a brain region that is thought to detect response conflict (Grinband et al., 2011). The link between response conflict and RT therefore appears to be a central feature of the conflict monitoring model. For this reason, even if RT-matched congruent and incongruent trials were not perfectly matched for conflict in the present face Stroop tasks (thereby explaining the presence of CSEs following RT-matched congruent and incongruent trials), the model would still have difficulty explaining why congruency effects were not smaller after slow-RT (high conflict) trials than after fast-RT (low conflict) trials in each congruency condition.

If CSEs are not driven by previous-trial response conflict (at least as operationalized by the conflict monitoring model), then which congruency-related processes might give rise to them? The view that CSEs are driven by expectancies regarding the congruency of the upcoming stimulus has received some support (Duthoo et al., 2013), but not in all studies (Alpay et al., 2009; Jiménez and Méndez, 2012). Recent studies also suggest that negative affect triggered by incongruent stimuli (van Steenbergen et al., 2010, 2012; Dreisbach and Fischer, 2011, 2012) and/or congruency switch costs stemming from the use of distinct processes on congruent and incongruent trials (Schmidt and De Houwer, 2011) may give rise to CSEs. Finally, it is possible that CSEs are driven by changes in a task's representation in working memory (Hazeltine et al., 2011). Future research should continue to develop and evaluate these potential sources of CSEs.

It is improbable for several reasons that the present findings stem from confounds related to exact stimulus or response repetitions (Mayr et al., 2003; Hommel et al., 2004). First, exact repetitions of the target face and/or the distracter word were not permitted in consecutive trials (Egner et al., 2010). Second, in Experiment 2, the frequencies of response repetitions and response alternations were equated across the four congruency sequences (Egner et al., 2010). Third, although the frequencies of response repetitions and response alternations could have differed across the four congruency sequences in the RT-matched data set, CSEs in the RT-matched data set did not differ from those in the full data set.

We acknowledge, however, that CSEs in the present tasks could reflect categorical repetitions of the distracter word (e.g., male -> MALE). Such repetitions differed in frequency across the four congruency sequences and might therefore have influenced the overall magnitude of CSEs. In theory, such an influence could have been mediated solely through feature integration processes or via an interaction between such processes and conflict-triggered control processes (Blais and Verguts, 2012). However, since this potential influence on CSEs was similar in the full data set, the RT-matched data set, and following slow-RT vs. fast-RT trials, it is unclear how it could have completely obscured an influence of previous-trial RT on current-trial congruency effects. Nevertheless, future studies will be required to determine the extent to which the present results generalize across such low-level task parameters, as well as across different distracter interference tasks. Indeed, Egner (2007) argued that CSEs reflect different mechanisms in different tasks (e.g., the Stroop, Simon, and Eriksen tasks). Thus, as when studying any aspect of CSEs, it is important to determine whether the results from one task generalize to others.

In sum, the present findings challenge the conflict monitoring model's account of CSE while offering novel support for congruency-based accounts. However, they are agnostic with regard to the specific congruency-related processes that drive CSEs. Future studies should therefore continue to investigate the psychological mechanisms underlying this phenomenon. Moreover, future modeling investigations should either revise the conflict monitoring model to account for the present results or develop new computational models of strategic adaptation in distracter interference tasks.

### Conflict of interest statement

The authors declare that the research was conducted in the absence of any commercial or financial relationships that could be construed as a potential conflict of interest.

## References

[B1] Akçay Ç. Hazeltine E. (2011). Domain-specific conflict adaptation without feature repetitions. Psychon. Bull. Rev. 18, 505–511 10.3758/s13423-011-0084-y 21404129

[B2] Alpay G. Goerke M. Stürmer B. (2009). Precueing imminent conflict does not override sequence-dependent interference adaptation. Psychol. Res. 73, 803–816 10.1007/s00426-008-0196-9 19034499

[B3] Blais C. Verguts T. (2012). Increasing set size breaks down sequential congruency: evidence for an associative locus of cognitive control. Acta Psychol. 141, 133–139 10.1016/j.actpsy.2012.07.009 22964053

[B4] Botvinick M. M. Braver T. S. Barch D. M. Carter C. S. Cohen J. D. (2001). Conflict monitoring and cognitive control. Psychol. Rev. 108, 624–652 10.1037/0033-295X.108.3.624 11488380

[B5] Burle B. Possamai C. Vidal F. Bonnet M. Hasbroucq T. (2002). Executive control in the Simon effect: an electromyographic and distributional analysis. Psychol. Res. 66, 324–336 10.1007/s00426-002-0105-6 12466929

[B6] Carp J. Kim K. Taylor S. Fitzgerald K. Weissman D. (2010). Conditional differences in mean reaction time explain effects of response congruency, but not accuracy, on posterior medial frontal cortex activity. Front. Hum. Neurosci. 4:231 10.3389/fnhum.2010.00231 21212836 PMC3014651

[B7] Compton R. Huber E. Levinson A. Zheutlin A. (2012). Is “conflict adaptation” driven by conflict? Behavioral and EEG evidence for the underappreciated role of congruent trials. Psychophysiology 49, 583–589 10.1111/j.1469-8986.2012.01354.x 22332754

[B8] Dreisbach G. Fischer R. (2011). If it's hard to read … try harder! Processing fluency as signal for effort adjustments. Psychol. Res. 75, 376–383 10.1007/s00426-010-0319-y 21210144

[B9] Dreisbach G. Fischer R. (2012). Conflicts as aversive signals. Brain Cogn. 78, 94–98 10.1016/j.bandc.2011.12.003 22218295

[B10] Duthoo W. Wuhr P. Notebaert W. (2013). The hot-hand fallacy in cognitive control: repetition expectancy modulates the congruency sequence effect. Psychon. Bull. Rev. 20, 798–805 10.3758/s13423-013-0390-7 23371807

[B11] Egner T. (2007). Congruency sequence effects and cognitive control. Cogn. Affect. Behav. Neurosci. 7, 380–390 10.3758/CABN.7.4.380 18189011

[B12] Egner T. (2008). Multiple conflict-driven control mechanisms in the human brain. Trends Cogn. Sci. 12, 374–380 10.1016/j.tics.2008.07.001 18760657

[B13] Egner T. Ely S. Grinband J. (2010). Going, going, gone: characterizing the time-course of congruency sequence effects. Front. Psychol. 1:154 10.3389/fpsyg.2010.00154 21833220 PMC3153769

[B14] Egner T. Etkin A. Gale S. Hirsch J. (2008). Dissociable neural systems resolve conflict from emotional versus nonemotional distracters. Cereb. Cortex 18, 1475–1484 10.1093/cercor/bhm179 17940084

[B15] Gratton G. Coles M. G. Donchin E. (1992). Optimizing the use of information: strategic control of activation of responses. J. Exp. Psychol. Gen. 121, 480–506 10.1037/0096-3445.121.4.480 1431740

[B16] Grinband J. Savitskaya J. Wager T. Teichert T. Ferrera V. Hirsch J. (2011). The dorsal medial frontal cortex is sensitive to time on task, not response conflict or error likelihood. Neuroimage 57, 303–311 10.1016/j.neuroimage.2010.12.027 21168515 PMC3114292

[B17] Hazeltine E. Lightman E. Schwarb H. Schumacher E. H. (2011). The boundaries of sequential modulations: evidence for set-level control. J. Exp. Psychol. Hum. Percept. Perform. 37, 1898–1914 10.1037/a0024662 21767054

[B18] Hommel B. Proctor R. Vu K.-P. (2004). A feature-integration account of sequential effects in the Simon task. Psychol. Res. 68, 1–17 10.1007/s00426-003-0132-y 14752663

[B19] Jiménez L. Méndez A. (2012). It is not what you expect: dissociating conflict adaptation from expectancies in a Stroop task. J. Exp. Psychol. Hum. Percept. Perform. 39, 271–284 10.1037/a0027734 22428671

[B20] Kerns J. Cohen J. MacDonald A. Cho R. Stenger A. Carter C. (2004). Anterior cingulate conflict monitoring and adjustments in control. Science 303, 1023–1026 10.1126/science.1089910 14963333

[B21] Lamers M. Roelofs A. (2011). Attentional control adjustments in Eriksen and Stroop task performance can be independent of response conflict. Q. J. Exp. Psychol. 64, 1056–1081 10.1080/17470218.2010.523792 21113864

[B22] Mayr U. Awh E. Laurey P. (2003). Conflict adaptation effects in the absence of executive control. Nat. Neurosci. 6, 450–452 10.1038/nn1051 12704394

[B23] Nieuwenhuis S. Stins J. Posthuma D. Polderman T. Boomsma D. de Geus E. (2006). Accounting for sequential trial effects in the flanker task: conflict adaptation or associative priming? Mem. Cogn. 34, 1260–1272 10.3758/BF03193270 17225507

[B24] Notebaert W. Verguts T. (2007). Dissociating conflict adaptation from feature integration: a multiple regression approach. J. Exp. Psychol. Hum. Percept. Perform. 33, 1256–1260 10.1037/0096-1523.33.5.1256 17924821

[B25] Schmidt J. De Houwer J. (2011). Now you see it, now you don't: controlling for contingencies and stimulus repetitions eliminates the Gratton effect. Acta Psychol. 138, 176–186 10.1016/j.actpsy.2011.06.002 21745649

[B26] Ullsperger M. Bylsma L. Botvinick M. (2005). The conflict adaptation effect: it's not just priming. Cogn. Affect. Behav. Neurosci. 5, 467–472 10.3758/CABN.5.4.467 16541815

[B27] van Steenbergen H. Band G. Hommel B. (2010). In the mood for adaptation: how affect regulates conflict-driven control. Psychol. Sci. 21, 1629–1634 10.1177/0956797610385951 20943936

[B28] van Steenbergen H. Band G. Hommel B. (2012). Reward valence modulates conflict-driven attentional adaptation: electrophysiological evidence. Biol. Psychol. 90, 234–241 10.1016/j.biopsycho.2012.03.018 22504294

[B29] Verbruggen F. Notebaert W. Liefooghe B. Vandierendonck A. (2006). Stimulus- and response-conflict-induced cognitive control in the flanker task. Psychon. Bull. Rev. 13, 328–333 16893003 10.3758/bf03193852

[B30] Wühr P. (2005). Evidence for gating of direct response activation in the Simon task. Psychon. Bull. Rev. 12, 282–288 10.3758/BF03196373 16082807

[B31] Yeung N. Botvinick M. Cohen J. (2004). The neural basis of error detection: conflict monitoring and the error-related negativity. Psychol. Rev. 111, 931–959 10.1037/0033-295X.111.4.931 15482068

[B32] Yeung N. Cohen J. Botvinick M. (2011). Errors of interpretation and modeling: a reply to Grinband et al. Neuroimage 57, 316–319 10.1016/j.neuroimage.2011.04.029 21530662 PMC3737739

